# Variability in C-type lectin receptors regulates neuropathic pain-like behavior after peripheral nerve injury

**DOI:** 10.1186/1744-8069-10-78

**Published:** 2014-12-10

**Authors:** Cecilia A Dominguez, Karl E Carlström, Xing-Mei Zhang, Faiez Al Nimer, Rickard P F Lindblom, Andre Ortlieb Guerreiro-Cacais, Fredrik Piehl

**Affiliations:** Department of Clinical Neuroscience, Neuroimmunology Unit, CMM L8:05, Karolinska Institutet, Stockholm, Sweden

**Keywords:** Neuropathic pain, Genetic, C-type lectin, Immune system, Microglia, Spinal cord, C-type lectin receptors regulate neuropathic pain by increased immune response in the spinal cord

## Abstract

**Introduction:**

Neuropathic pain is believed to be influenced in part by inflammatory processes. In this study we examined the effect of variability in the C-type lectin gene cluster (Aplec) on the development of neuropathic pain-like behavior after ligation of the L5 spinal nerve in the inbred DA and the congenic Aplec strains, which carries seven C-type lectin genes originating from the PVG strain.

**Results:**

While both strains displayed neuropathic pain behavior early after injury, the Aplec strain remained sensitive throughout the whole study period. Analyses of several mRNA transcripts revealed that the expression of Interleukin-1β, Substance P and Cathepsin S were more up-regulated in the dorsal part of the spinal cord of Aplec rats compared to DA, indicating a stronger inflammatory response. This notion was supported by flow cytometric analysis revealing increased infiltration of activated macrophages into the spinal cord. In addition, macrophages from the Aplec strain stimulated *in vitro* displayed higher expression of inflammatory cytokines compared to DA cells. Finally, we bred a recombinant congenic strain (R11R6) comprising only four of the seven Aplec genes, which displayed similar clinical and immune phenotypes as the Aplec strain.

**Conclusion:**

We here for the first time demonstrate that C-type lectins, a family of innate immune receptors with largely unknown functions in the nervous system, are involved in regulation of inflammation and development of neuropathic pain behavior after nerve injury. Further experimental and clinical studies are needed to dissect the underlying mechanisms more in detail as well as any possible relevance for human conditions.

## Introduction

Injuries to either the peripheral or central nervous systems (CNS) often lead to chronic neuropathic pain conditions. The underlying mechanisms are not clarified in detail, hence therapeutic options are limited. However immune related reactions in the nervous system are suggested to be of importance both for the maintenance and development of neuropathic pain [1, 2]. One such feature is the recruitment of leukocytes into the CNS after a peripheral nerve injury, which may amplify or modify the inflammatory activation of CNS resident glial cells, in turn leading to exaggerated pain [3–6]. Thus, infiltration of blood monocyte-derived macrophages, is an early phenomenon upon nerve injury. Although involved in the clearance of debris due to their phagocytic properties, activated macrophages also release a range of cytokines and chemokines, which have been linked to pain-related behavior [7–9]. In previous studies the chemokine ligand 2 (Ccl2)- chemokine receptor 2 (Ccr2) signaling has been shown to be critically important for the attraction of monocytes to the CNS, which is in turn of relevance for development of neuropathic pain [6]. Also, other types of leukocytes, including T-cells have been suggested to be involved in neuropathic pain-like behavior [5, 10].

Previous studies on inbred rat and mice strains suggest a considerable genetic contribution to various experimental pain phenotypes [11–14]. However, knowledge of exactly defined molecular pathways involved in, or if genetic influence acts on regulation of inflammatory processes of relevance for neuropathic pain, is limited. In an earlier study we could demonstrate that the MHC locus, a region of about 200 genes, exerts a significant effect on pain susceptibility in inbred rat strains after a peripheral nerve injury [15]. Interestingly, we recently replicated this finding in humans by showing that carriers of the HLA DQB1*03:02 allele displayed an increased risk of developing a neuropathic pain condition after a peripheral nerve lesion [16]. The mechanisms underlying this genetic effect are still unclear, but effects on nerve injury-induced immune reactions are likely given the role of the MHC in these contexts.

To further examine the role of genetically regulated immune reactions for pain susceptibility after nerve injury we here investigated the effect of a small rat chromosome 4 gene fragment containing seven C-type lectin receptors (CLRs). The gene cluster, denoted antigen-presenting lectin-like receptor gene complex (Aplec), has previously been studied primarily in models of autoimmune and infectious disease, where it has been demonstrated to regulate different aspects of the innate immune response [17, 18]. Also, we recently found the Aplec cluster to regulate the immune phenotype after a mechanical ventral root injury, including effects on leukocyte recruitment [19]. The aim here was to explore the importance of variability in the Aplec cluster occurring among inbred rat strains for neuropathic pain-like behavior and immune phenotype after a standardized spinal nerve injury.

## Results

The Aplec strain is susceptible to develop neuropathic-pain like behavior.

**Figure 1:**
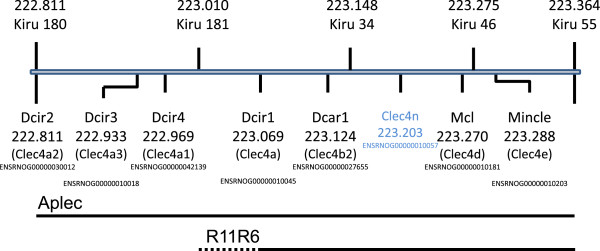
**Schematic map of the seven Aplec genes and the four genes with in R11R6 strain on chromosome 4 (222.811-223.365 kb respectively 223.010-223.365 kb).** The microsatellite markers are depicted in the upper part. Clec4n is a pseudogene. (Modified figure from Ensembl version 73, Rnor 5.0).

**Figure 2:**
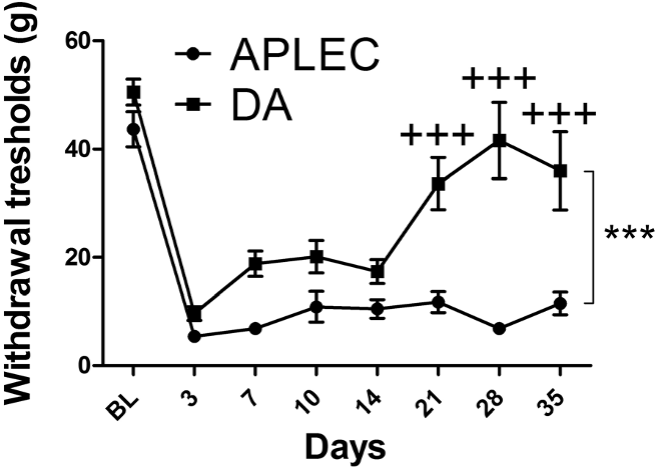
**Neuropathic pain-like behavior after peripheral nerve injury in DA and Aplec strain.** One-way ANOVA demonstrates overall differences between the strains (***p < 0.001). Bonferroni *post-hoc* testing reveals significant difference between DA and Aplec on days 21, 28 and 35 (+++p < 0.001), Data are expressed as means ± SEM. (12–20 rats per group).

DA and Aplec rats are genetically identical except that the latter contains a small genetic fragment from the PVG strain comprising seven genes, all of which are CLRs (Figure 1) [18, 20, 21]. We here tested susceptibility to develop neuropathic pain behavior in a SNL model. Initially, both strains developed mechanical hypersensitivity to a similar degree after SNL, however, DA rats had started to recover after 14 days, whereas the Aplec rats remained sensitive during the entire time of testing (Figure 2). Statistical analysis demonstrated overall differences between the strains, as well as statistically significant differences specifically on day 21, 28 and 35, the last three time points that were tested.

### Expression of Aplec genes

**Figure 3:**
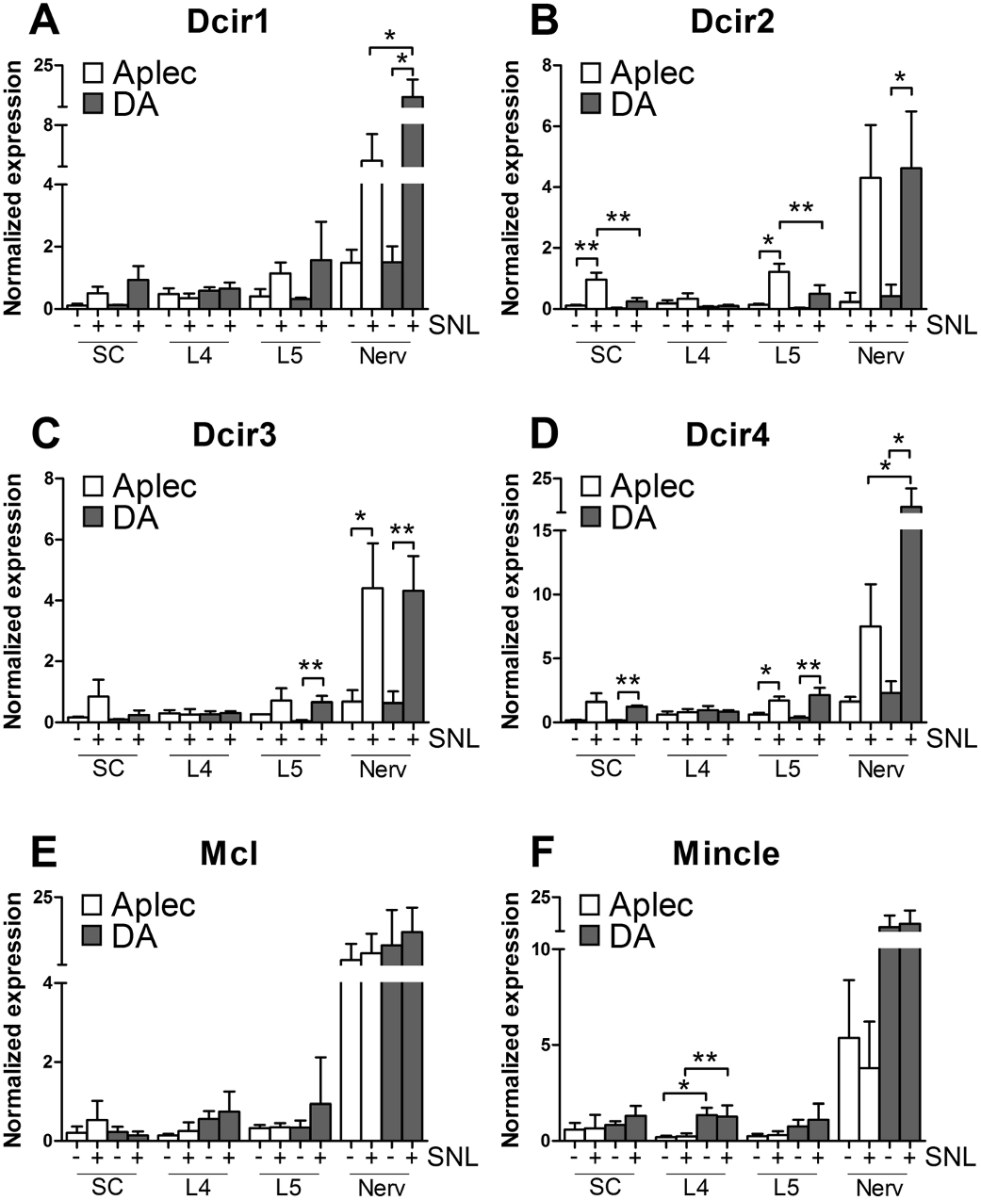
**Expression levels of Dcir1-4, Mcl and Mincle detected in spinal cord, L4 DRG, L5 DRG and nerve.** mRNA levels were analyzed by RT-PCR in Aplec and DA strain 7 days after injury (+) and in healthy controls (−) from each strain. Dcir1 **(A)**, Dcir2 **(B)**, Dcir3 **(C)**, Dcir4 **(D)**, Mcl **(E)**, Mincle **(F)**. One-way ANOVA was done followed by Step-down Bonferroni correction for multiple comparisons (*p < 0.05; **p < 0.01).

In order to study any possible expression differences of the seven CLRs in the Aplec fragment the three anatomical locations primarily affected by the injury, i.e. SC, DRG and nerve, were analyzed by RT-PCR. In general, the injury induced expression in the studied tissues was most pronounced in the peripheral nerve, where Dcir1-4 all were increased on both strains (Figure 3A-D). Also in the L5 DRG and the SC these four transcripts were up-regulated after injury but to a lower degree. In contrast, Mcl and Mincle were not significantly affected by injury in any of the locations (Figure 3E-F). In the comparison between strains, Dcir1 and 4 were higher in Aplec compared to DA in peripheral nerve, while Dcir2 was higher in L5 DRG and SC of DA rats. The expression of Dcar1 was below detection limit (data not shown).

### Expression of neuropeptides, cytokines and chemokines receptors

**Figure 4:**
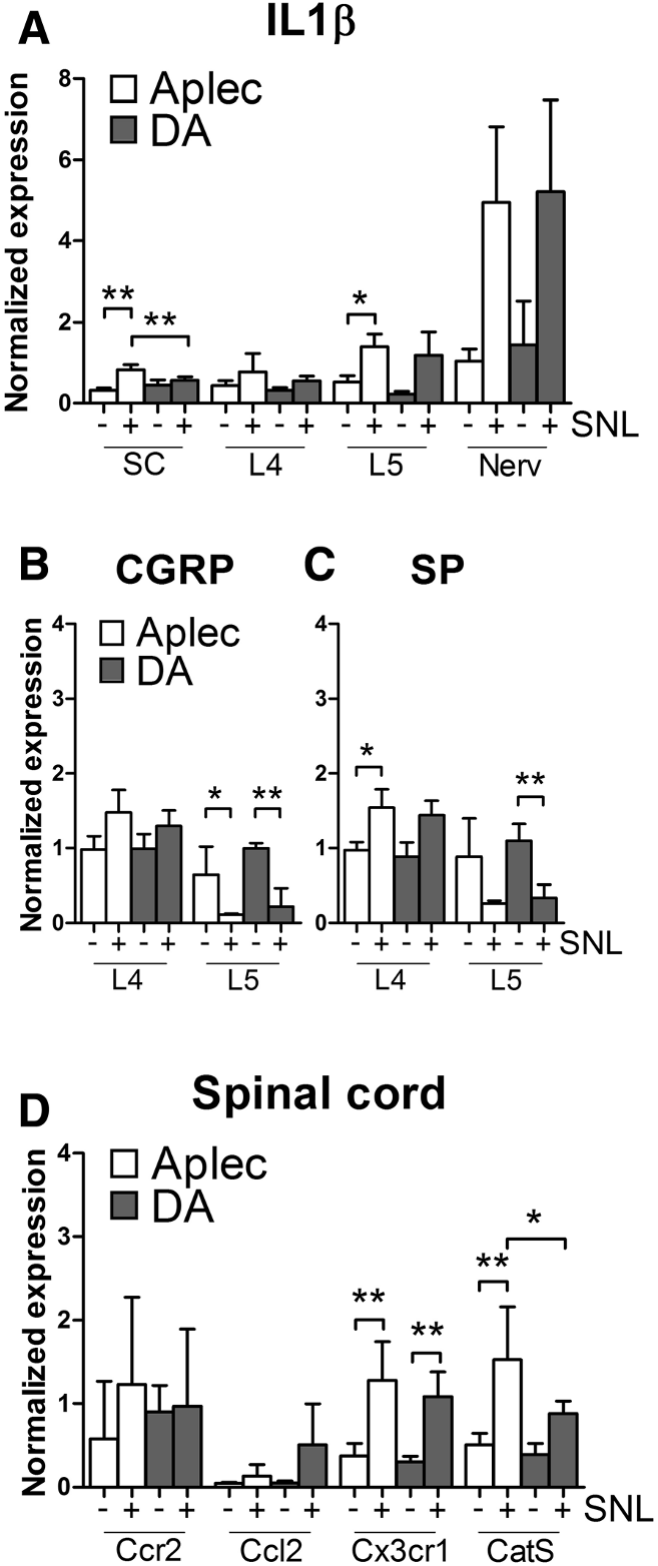
**Expression levels of pain molecules in spinal cord, L4 DRG, L5 DRG and nerve 7 days after injury (+) and health controls (−) in Aplec and DA rats.** IL-1β **(A)**, CGRP L4 and L5 DRG **(B)**, SP L4 and L5 DRG **(C)**, Ccr2, Ccl2, Cx3cr1 and CatS in SC **(D)**. Statistical analysis were done with one-way ANOVA and by Step-down Bonferroni correction for multiple comparison (*p < 0.05; **p < 0.01).

The cascade of events occurring after a peripheral nerve lesion includes changes in the expression of neuropeptides, cytokines and chemokines. We therefore measured the expression of Substance P (SP) and Calcitonin gene-related peptide (CGRP), Interleukin-1β (IL1β), Ccr2, Fractalkine receptor (Cx3cr1) and Cathepsin S (CatS). As expected, there was up-regulation of IL-1β expression in all studied tissues (Figure 4A). SP and CGRP were down-regulated in the lesioned L5 DRG, with a trend for higher expression in the adjacent intact L4 DRG (Figure 4B-C). Ccr2, Ccl2, Cx3cr1 and CatS were only studied in the SC, where the latter two were up-regulated (Figure 4D). The expression of IL1β and CatS were higher in SC of Aplec compared to DA rats. Ccr2 and Ccl2 levels were not affected after injury in either of the strains (Figure 4D).

### Immune cell infiltration in the spinal cord

**Figure 5:**
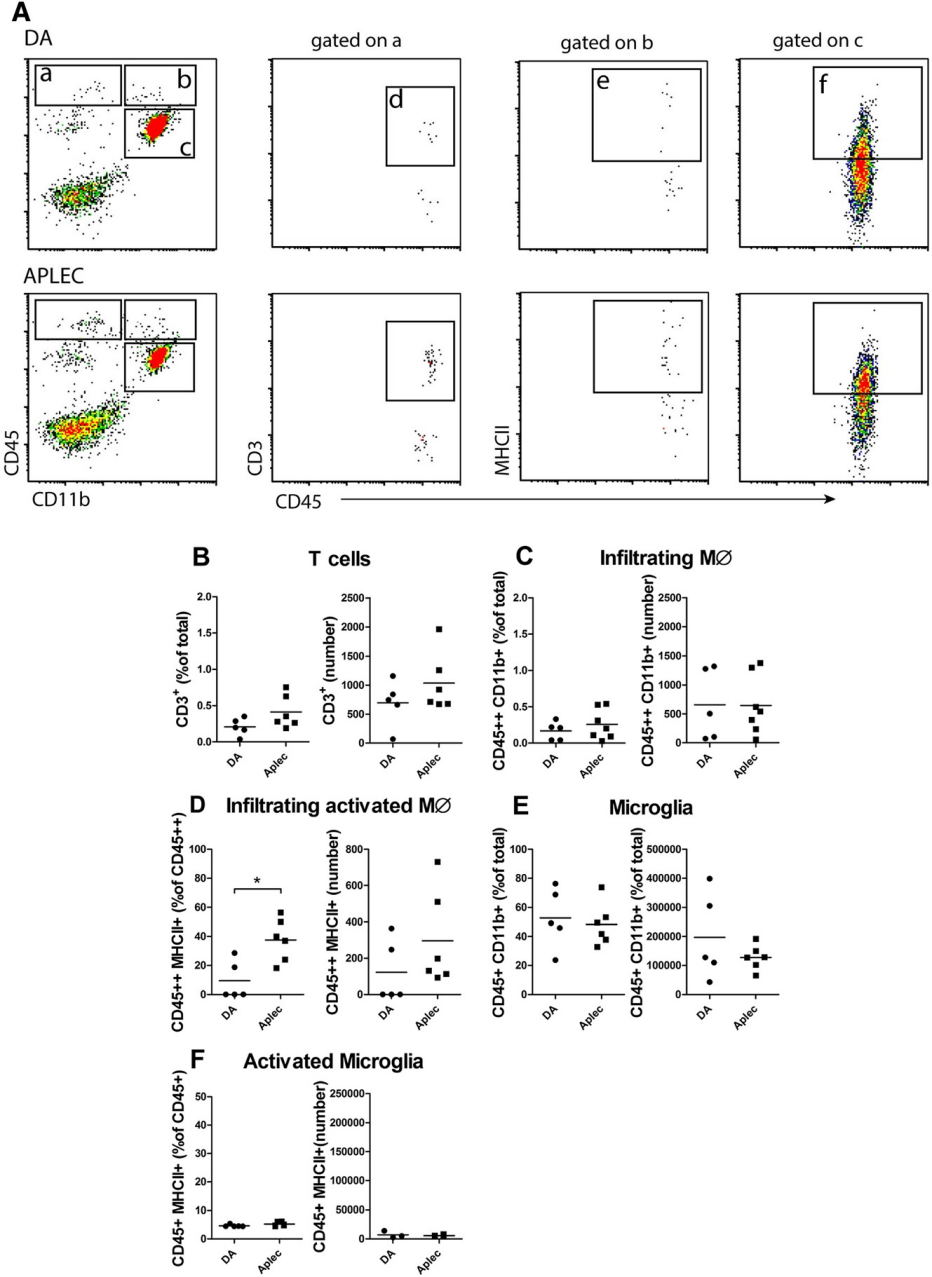
**Immune cell infiltration to the spinal cord.** Flow cytometry analyses performed 14 days after injury in Aplec and DA strains. Representative flow cytometry plots **(A)**. Gated areas represent different cell populations, lymphocytes **(a)**, macrophages **(b)**, microglia **(c)** T-cells **(d)** activated infiltrating macrophages **(e)** and activated microglia **(f)**. Strain differences are presented as percent of total cells and absolute numbers for T-cells **(B)**, infiltrating macrophages **(C)**, activated infiltrating macrophages **(D)**, microglia **(E)** and activated microglia **(F)**. Statistical analysis were done by Mann–Whitney test (*p < 0.05).

Flow cytometry analyses were performed in DA and Aplec, 14 days post-injury to characterize immune cell recruitment to the SC. Populations of microglia, monocyte/macrophages, T-cells and NK cells were analyzed (Figure 5A). As expected, the microglia population greatly outnumbered the other two. There was a tendency for a higher relative proportion of both microglia and T-cells in Aplec compared to DA. In addition, the Aplec strain had a significantly increased proportion of infiltrating activated macrophages (CD45^+^MHCII^+^) compared to DA (Figure 5B-F).

### *In vitro*stimulation of bone marrow derived macrophages

**Figure 6:**
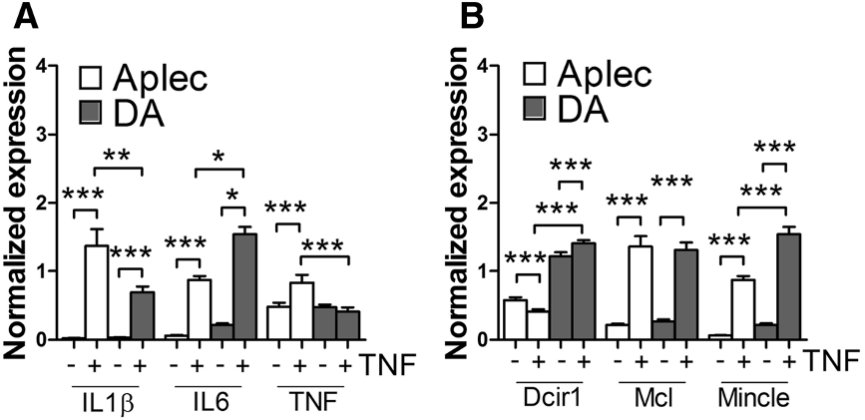
**Expression levels of pro-inflammatory cytokines, Dcir1, Mincle and Mcl in BMMφ from DA and Aplec following stimulation with TNF- α.** The Aplec strain display higher expression of TNF -α , IL-6 and IL-1β **(A)**. Dcir1 and Mincle levels are higher in DA strain after stimulation, Mcl levels are up-regulated in both strains after stimulation but without strain differences **(B)**. One-way ANOVA followed by Bonferroni *post-hoc* (*p < 0.05; **p < 0.01; ***p < 0.001).

CLRs are expressed by antigen-presenting cells, like macrophages. As we observed an increased infiltration of macrophages to the SC after injury we performed *in vitro* stimulations of macrophages from both stains to determine possible phenotypic differences in the expression of pro-inflammatory cytokines. Accordingly, bone marrow-derived macrophages (BMMφ) from DA and Aplec rats were stimulated *in vitro* with TNFα for 24 h. We could detect a significant difference in expression levels of IL-1β, IL-6 and TNFα, where BMMφ cells derived from the Aplec strain expressed higher levels compared to DA (Figure 6A). Interestingly, Dcir1 and Mincle levels were higher in DA strain after stimulation compared to Aplec. Mcl levels were up-regulated after stimulation in both strains but without strain differences (Figure 6B).

### Neuropathic pain-like behavior in the R11R6 congenic rat strain

**Figure 7:**
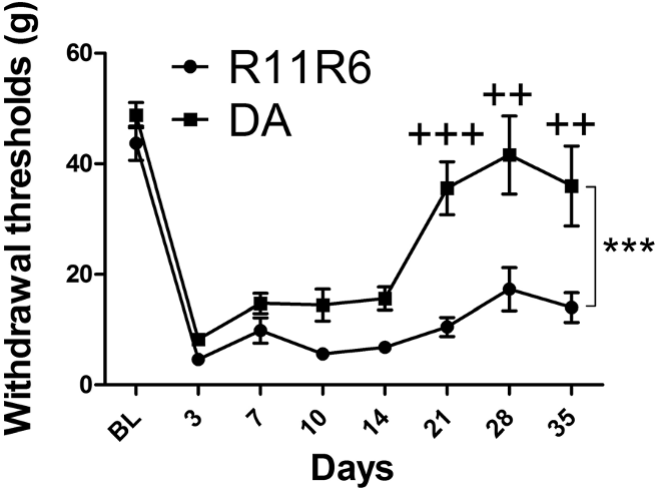
**Neuropathic pain-like behavior after peripheral nerve injury in DA and R11R6 strain.** One-way ANOVA demonstrates overall differences between the strains (***p < 0.001). Bonferroni *post-hoc* indicates significant difference between DA and R11R6 on day 21, 28 and 35 (++p < 0.01; +++p < 0.001) Data are expressed as means ± SEM. (12–20 rats per group).

**Figure 8:**
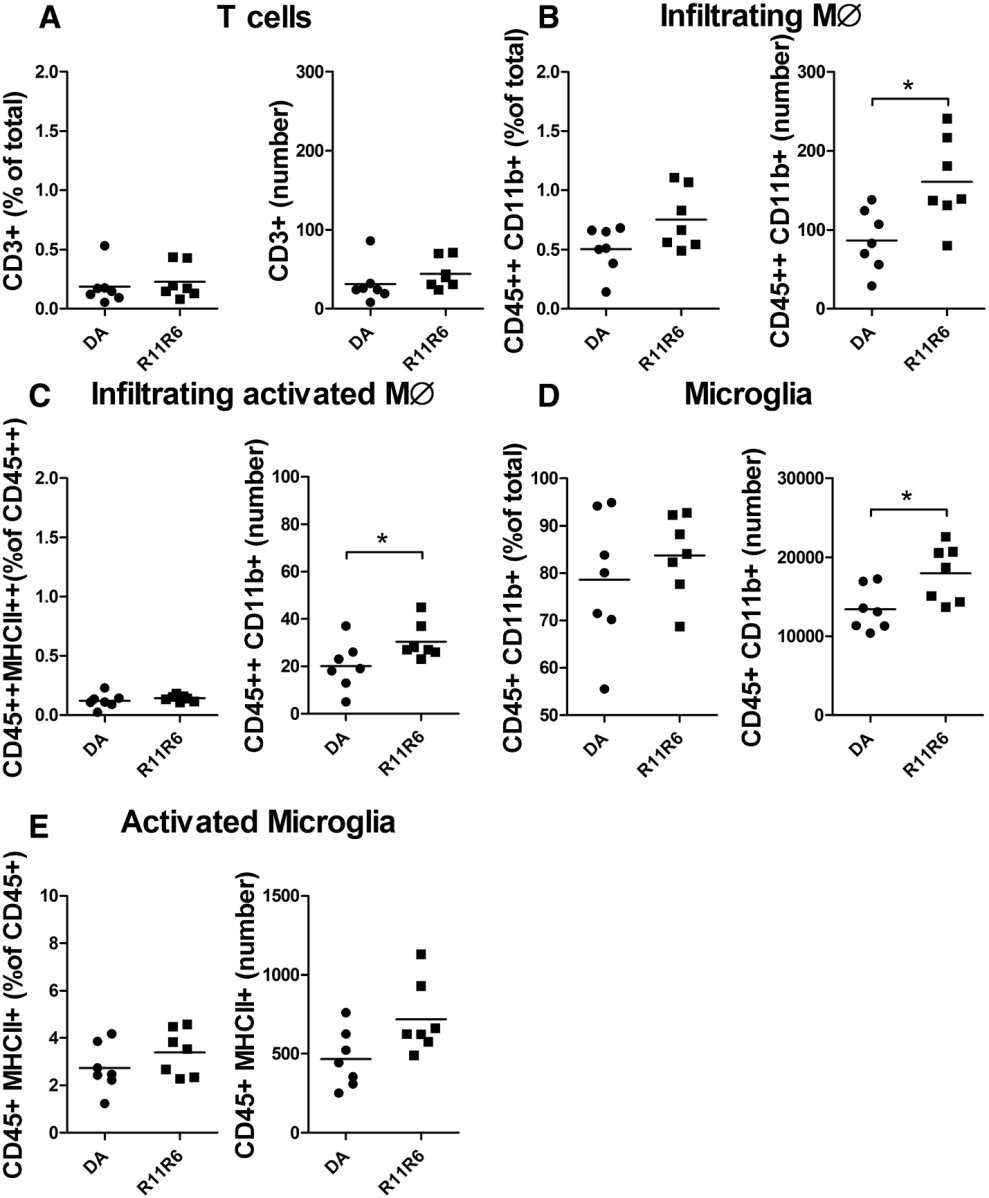
**Immune cell regulation to the spinal cord in DA and R11R6 strain 14 days after injury.** Flow cytometric analysis using gates as in Figure 5A. Strain differences are presented as percent of total cells and absolute numbers for T-cells **(A)**, infiltrating macrophages **(B)**, activated infiltrating macrophages **(C)**, microglia **(D)** and activated microglia **(E)**. Statistical analysis were done by Mann–Whitney test (*p < 0.05).

In order to reproduce our findings, and genetically dissect the Aplec cluster further, a smaller congenic containing only four out of the seven CLRs from the Aplec fragment; Dcir1, Dcar1, Mcl and Mincle, was created. Following SNL the R11R6 developed mechanical hypersensitivity to a similar extent as the Aplec strain and remained sensitive during the entire time of testing (Figure 7). As for Aplec, statistical analysis demonstrated overall differences between the strains, as well as significant differences specifically on day 21, 28 and 35, i.e. the last three time points tested. To assess the cellular phenotype in R11R6 flow cytometry analyse were performed as for the Aplec, however, including only the L4-L6 segment of the cord. As with the Aplec strain the R11R6 had more infiltration of activated macrophages compared to DA. In addition, also the absolute numbers of microglia and infiltrating macrophages were greater in R11R6 compared to DA (Figure 8A-E).

## Discussion

In the present study we demonstrate that the Aplec congenic rat displays a nerve injury phenotype distinctly different from DA rats, with continued neuropathic pain-behavior extending well after the DA strain has recovered. The phenotype is associated with increased expression of IL-1β and CatS, as well as increased infiltration of activated macrophages to the SC and a greater response to an inflammatory stimulus of BMMφ *in vitro*, well in line with the notion of an inflammatory component in neuropathic pain development. The seven CLRs comprised in the cluster are expressed by antigen-presenting cells as well as neutrophils [22, 23] and act as pattern recognition receptors that upon binding of a pathogen or endogenous ligand will shape T -cell responses and modulate the ensuing inflammatory reaction [22, 24, 25]. The Aplec cluster was originally position mapped by comparing the susceptibility of inbred DA rats with that of DA rats carrying alleles derived from the oil-induced arthritis-resistant PVG strain [18]. In a subsequent study the Aplec cluster was found to affect the *in vivo* and *in vitro* phenotypes with regard to infectious and inflammatory challenges, further strengthening the notion of effects mediated through the regulation of general macrophage activation status [17]. Interestingly, genetic variability in the corresponding human genes has been associated with susceptibility to anti-citrulline antibody negative rheumatoid arthritis, indicating relevance also for human disease [21].

The role of CLRs has mostly been studied in the context of antigen-presenting cells, in particular dendritic cells, where they have been shown to important for shaping adaptive immune responses [26, 27]. However, detailed knowledge of the molecular function of many CLRs is still lacking and any possible involvement of CLRs in traumatic nerve injuries is entirely unknown. However, in a recent expression quantitative trait loci mapping study we found Dcir3 to be significantly regulated in the SC between DA and PVG after ventral nerve root injury [19]. Interestingly, further testing of the Aplec strain in this injury model revealed an effect on the inflammatory response with more lymphocyte infiltration as well as increased survival of avulsed motoneurons. Here we find a different pattern of regulated genes in the Aplec cluster, with higher expression of Dcir 2 in both SC and L5 DRG of the Aplec strain. In contrast, in the nerve, Dcir 1 and 4 levels were higher in the DA strain. Mincle was not induced by injury, but expression was in general higher in the DA strain. Taken together this suggests that there are complex regulatory differences affecting the expression pattern of several of the CLRs in the fragment underlying the observed phenotypes.

By a large scale breeding effort we were able to identify a sub-congenic with a recombination within the Aplec cluster, isolating four of the seven CLRs in a new fragment; R11R6, containing Dcir1, Dcar1, Mcl and Mincle. Testing of this strain in the SNL model revealed a phenotype almost identical to Aplec, suggesting the underlying genetic variability or variabilities conferring the clinical effect to be localized to this fragment. Of the four genes in the R11R6 fragment Dcar1 could be viewed as a potential candidate since it is nonsense mutated in DA strain [21]. However, the mRNA levels of Dcar1 were barely detectable in all studied tissue arguing against a role for Dcar1. Mcl and Mincle were not induced by injury, but expression of Mincle was higher in the DA strain compared to the Aplec. The last gene, Dcir1, was expressed more highly in the nerve of DA rats and is known to contain an immunoreceptor tyrosine-based inhibitory motif, hence involved in inhibitory signaling [22, 28]. This may imply that DA up-regulate Dcir1 to inhibit activation/secretion of proinflammatory cytokines. *In vitro* stimulation of BMMφ with TNF-α resulted in higher levels of Dcir1 in DA cells. Given the genetic complexity with possible mutual cross-regulation between the genes in the fragment and differences between different anatomical locations, formal proof of the underlying causative genetic variation or variations may require continued recombinant inbred breeding, an undertaking that could take several years.

We further explored downstream molecular events segregating between the two studied strains. The finding that IL-1β levels were significantly higher in the pain sensitive strain in the SC compared to DA after injury in L5 DRG are in concordance with several studies demonstrating that IL-1β increases neuron excitability and accelerate central sensitization [10, 29]. Expression of IL-1β was also greater in i*n vitro* stimulated BMMφ, suggestin that the Aplec cluster affects expression of this cytokine. As expecte, expression of SP and CGRP was down regulated in both strains after injury, in accordance with pervious knowledge [30–32]. On the contrary we observed an up-regulation of SP and CGRP levels in both strains in the intact L4 DRG, with significantly higher expression of SP in the Aplec strain. Fukuoka et al. observed a similar finding with increased CGRP levels in the contralateral L4 DRG after same type of injury, which may reflect increased activity or sensitivity in intact sensory pain transmission systems, possibly including also inflammatory cytokines [33–36].

Ccl2/Ccr2 and Fractalkine/Cxcr1 are two signaling pathways known to be involved in mediating interaction between injured sensory neurons and microglia [6, 37], in addition Ccl2/Ccr2 signaling has been shown to be important for both monocyte recruitment and pain sensitivity [5, 6]. We could not observe any differences in the expression of Ccl2/Ccr2 in the SC, which could indicate either that the number of recruited macrophages is too low to be detected with this approach or that signaling through this ligand-receptor pair is of less importance in the context studied here. In contrast, we could record an up-regulation of Cx3cr1 in both strains after injury. Interestingly, CatS was up-regulated preferentially in the cord of the pain sensitive Aplec strain. CatS, a proteolytic lysosomal cysteine proteinase, is released by activated microglia in SC and macrophages in the periphery and is responsible for the cleavage of Fractalkine which gives rise to a soluble cleavage product that binds to Cx3cr1 expressing microglia leading to an enhancement of pro-nociceptive mediators [38, 39].

The role of T-cells for the development of neuropathic-pain like behavior is complex, with contrasting effects including both pain-driving and analgesic effects [3, 40–42]. In a previous study of a motor nerve avulsion model we found a greater T -cell infiltration to the SC in Aplec compared to DA [19]. Here we found a tendency for both Aplec and R11R6 rats to have higher numbers of T-cells compared to DA. In contrast, strain differences were evident for infiltration of activated macrophages in Aplec and subsequently confirmed in the R11R6 strain, displaying also increased numbers of microglia as well as macrophages in general.

Previous reports have demonstrated that infiltration of BMMφ to the CNS play a role in the development of neuropathic pain [6]. Hence, the production of cytokines by activated BMMφ cells was examined *in vitro* using a standard inflammatory stimulus. We could detect that cells derived from the pain sensitive Aplec strain displayed higher expression of TNF-α, IL-1β and IL-6, all of which are known to increase both pain sensitivity and induces the production of each other, which amplifies the inflammatory response [7, 43]. This is in line with a previous study suggesting the Aplec cluster to regulate the general activation status of macrophages [17].

## Conclusion

All together our findings support the conclusion that variability in CLRs occurring among inbred rat strains affects inflammatory activation of antigen-presenting cells, with subsequent effects on pain transmitting systems. Importantly, our results, derived from large scale genetic dissection, identified that variability in the four CLR’s in the R11R6 sub-congenic (Dcir1, Dcar1, Mcl and Mincle) is sufficient to cause a significant difference in the clinical effect. Further studies are needed to elucidate the mechanisms more in detail. The fact that this gene cluster was identified by unbiased forward genetics and that genetic variability in human orthologuos have been associated with disease risk encourage studies also in humans.

## Materials and methods

### Ethics statement

All animal experiments were performed in accordance with the Guidelines of the International Association for the Study of Pain and were approved by the Swedish ethical committee (Stockholm’s North Ethical Committee- Stockholms Norra Djurförsöksetiska nämnd).

### Animals

Two congenic rat strains, one containing seven CLR genes denoted antigen-presenting lectin-like receptor complex (Aplec) and the other containing four CLR genes denoted R11R6, as well as the inbred Dark agouti (DA) male rats were used in this study. The congenic Aplec and R11R6 were produced by transferring a gene cluster from Piebald Virol Glaxo (PVG) rats onto Dark-Agouti (DA) rats through repeated backcrossing as previously described [18]. The fragments are on chromosome 4 (222.811-223.365 kb respectively 223.010-223.365 kb) and a schematic map with the gene positions (Gene ID) and markers are depicted in Figure 1 (Ensembl version 73, Rnor 5.0).

All animals were kept under specific pathogen-free and climate-controlled conditions with 12 h light/dark cycles, housed in polystyrene cages containing wood shavings, and fed standard rodent chow and water *ad libitum*.

### Peripheral nerve injury

Rats were subjected to modified spinal nerve ligation model (SNL) [44] under standardized conditions. The animals were deeply anesthetized with 2% isoflurane and lower back skin was shaved and cleaned with 70% ethanol. An incision was made through the skin and paraspinal muscle were separated from the spinous processes at the L5-L6 levels. The fifth lumbar spinal nerve was transected distal to the ganglion. The skin was closed in layers and sutured. 0,25 ml Eusaprim (16 mg/ml sulfametoxazol, 80 mg/ml trimethoprim) (Aspen Europé Gmbh, Bad Oldesloe, Germany) was administrated post surgery, subcutaneously. All rats were sacrificed with CO2 and perfused with PBS containing Heparin (LEO, Pharma AB, Malmö, Sweden). Rats were sacrificed at day 7,14 and 35 after injury.

### Behavioral testing

Rat were tested for mechanical hypersensitivity before and on day 3,7,10 and 14 after injury, and then weekly at week 3 and 4. Individual rats were placed in testing chambers with metal mesh floor 10 min before experiments for habituation. A set of calibrated nylon monofilament (Semmes-Weinstein monofilaments, Stoelting, IL) was applied to the glabrous skin of the paws with increasing force until the animal withdrew the limb. Each monofilament was applied 5 times with a few seconds interval and withdrawal threshold was determined when the rat withdrew the paw from at least 3 out of 5 stimulations.

### Quantitative real-time PCR (qPCR)

The ipsilateral L5 and adjacent unlesioned L4 dorsal root ganglion (DRG) were identified using a dissection microscope and taken for subsequent analysis. Also the ipsilateral dorsal horn of the spinal cord (SC) (segment L4-L5) and a few millimeter of the nerve proximal to the injury were collected for mRNA quantification at day 7 after SNL. Total RNA was extracted with RNeasy Mini kit (Qiagen) and RNase-Free DNase Set (Qiagen) according to manufacturer’s protocols. cDNA was prepared with 5x iScript reaction mix (Bio-Rad) with 5 μl total RNA. Amplifications were conducted using Bio-Rad SYBR green according to manufacturer’s instructions and plates were run in Bio-Rad CFX optical system (Bio-Rad). Primer specificity was assessed by determining amplicon size using gel electrophoresis and melt curve analysis of each reaction indicating a single peak. The targets analyzed and their primer sequences are listed in Table 1. Normalized expressions were calculated in Bio Rad CFX manager v2.0 (Bio-Rad) using *hprt* and *gapdh* as house-keeping genes.

**Table 1:** **Sequences of primers used for RT-PCR**

Target name	Forward sequence	Reverse sequence
Gapdh	TCA ACT ACA TGG TCT ACA TGT TCC AG	TCC CAT TCT CAG CCT TGA CTG
Hprt	CTC ATG GAC TGA TTA TGG ACA	GCA GGT CAG CAA AGA ACT TAT
SP	TGG CGG TCT TTT TTC TCG TT	GCA TTG CCT CCT TGA TTT GG
CGRP	GTG TCA CTG CCC AGA AGA GAT C	CAA AGT TGT CCT TCA CCA CAC C
IL-1β	GAAAGACGGCACACCCACC	AAACCGCTTTTCCATCTTCTTCT
Dcir1	CCATAGCAAGGAAGAACAGGACTT	TGAATCCCAGAGCCCTATAAAATAA
Dcir2	CCATCATCCAAGTAAGCCAGGTTC	GAGTCAGTTGAAGTAAAGTAGCAGTAG
Dcir3	TGCCACAAGTTCTCCAAG	TCCAATTCAGTATAGTTCAGTTCC
Dcir4	CATTCGTCCGTGGAAGACAAA	TGCAGAGTCCCTGGAAGTGAA
Dcar1	TGCTCATCTGTTGGTGATCCA	TGTAAAATAACCCCAACGAGTGTCTA
Mcl	CACAAGGCTAACATGCATCCTAGA	GCAAAGTAACAGTTAGACTGGAATGCT
Mincle	TTTCACAGAGTCCCTGAGCTTCT	TCCCTCATGGTGGCACAGT
TNF-α	GACCCTCACACTCAGATCCAGATCATCTTCT	ACGCTGGCTCAGCCACTC
Ccr-2	AGAAGTATCCAAGAGCTTGATGAGG	ATAGTGAGCCCAGAATGGGAG
Ccl-2	AACTCTCACTGAAGCCAGAT	GGTGACAAATACTACAGCTTC
Cx3cr1	CTGCTCAGGACCTCACCAT	CAGACCGAACGTGAAGACAA
Cathepsin S	TGT TCT CGT GGT TGG CTA T	AAC GGT TTA GAT TTC TGG GT
IL-6	AGAAAAGAGTTGTGCAATGG	ACAAACTCCAGGTAGAAACG

### Flow cytometry

At day 14 after injury animals were scarified with CO2 and perfused through the ascending aorta with ice-cold PBS supplemented with heparin (LEO Pharma AB, Malmö, Sweden). The spinal cords (n = 5-7 rats/strain) were removed and homogenized with a glass tissue grinder in a 50% Percoll solution (Sigma-Aldrich, Stockholm, Sweden). A density gradient was made consisting of the following layers: a top layer of 30% Percoll (20 ml), a middle layer with the homogenized tissue in 50% Percoll (20 ml) and a bottom layer of 63% Percoll (7 ml). All Percoll solutions were made fresh by diluting Percoll in 10xHBSS (Hank’s Balanced Salt Solution, Gibco), supplemented with 0.1% BSA and 0.1% glucose. After centrifugation at 1000 g at 7°C for 30 min, cells below the myelin layer were collected, washed with PBS containing 0.5% FBS and 2 mM EDTA and stained with the following antibodies: CD3-FITC, MHCII-PerCP, CD45-APC and CD11b-APC-Cy7 (eBioscience). Samples were run in Gallios flow cytometer (Beckman Coulter, Brea, USA) and analysis of acquired cells was performed with Kaluza v1.1 (Beckman Coulter). In the first experiment done on Aplec and DA rats the whole spinal cord was taken for analysis whereas for the R11R6 and DA experiment only the lumbar segment of interest, L4-L6 was taken for analysis.

### Bone marrow-derived macrophages culture

Bone marrow-derived macrophages were cultured as described previously [45] from naive DA and Aplec rats. In brief, femurs were dissected and femoral bone-marrow cells were collected by flushing through medium with a 21-gauge needle. Single-cell suspensions were prepared and re-suspended in Dulbecco’s modified Eagle’s medium (DMEM) (Gibco) supplemented with 20% heat-inactivated FBS, 100 U/ml penicillin, 100 μg/ml streptomycin, 2 mM L-glutamine and 20% M-CSF conditioned L929 cell line supernatant. Bone marrow cells were cultured in 175 cm^2^ cell culture flasks and incubated at 37°C and 5% C0_2_ in a humidified incubator for 10 days; medium was changed after 4 and 6 days with M-CSF conditioned medium, and after 10 days with complete medium (DMEM + FBS) without M-CSF conditioning. Cells were harvested using EDTA (Sigma) at a concentration of 0.5 mM and seeded in 24-well plates (4 × 10^5^ cells/well).

The cells were then left un-stimulated, or stimulated with TNF-α (20 ng/ml) for 24 h and then taken for analysis with RT-PCR.

### Statistical analysis

Statistical analyses were conducted using Graphpad Prism (5.0). For behavioral analysis one-way ANOVA data analysis were performed for overall differences (***p < 0.001) followed by Bonferroni *post-hoc* for individual time points (++p < 0.01; +++p < 0.001). Data are expressed as means ± standard error of the mean. For in vivo RT-PCR analysis one-way ANOVA was done followed by Step-down Bonferroni correction for multiple comparisons. The in vitro RT-PCR analysis were done by one way ANOVA followed by Bonferroni *post-hoc* (*p < 0.05; **p < 0.01; ***p < 0.001). The flow cytometry studies were analysed with Mann–Whitney test (*p < 0.05).
